# Development process of a clinical guideline to manage type 2 diabetes in adults by Ayurvedic practitioners

**DOI:** 10.3389/fmed.2023.1043715

**Published:** 2023-01-30

**Authors:** Kaushik Chattopadhyay, Nitin Kapoor, Michael Heinrich, Achintya Mitra, Madhukar Mittal, Sarah Anne Lewis, Sheila Margaret Greenfield, Shyamalendu Mukherjee, Ivo Pischel, Panniyammakal Jeemon, Nikhil Tandon, Sanjay Kinra, Tuhin Kanti Biswas, Jo Leonardi-Bee

**Affiliations:** ^1^Lifespan and Population Health Academic Unit, School of Medicine, University of Nottingham, Nottingham, United Kingdom; ^2^The Nottingham Centre for Evidence-Based Healthcare: A JBI Centre of Excellence, Nottingham, United Kingdom; ^3^Department of Endocrinology, Metabolism and Diabetes, Christian Medical College, Vellore, India; ^4^Non-communicable Diseases and Implementation Science, Baker Heart and Diabetes Institute, Melbourne, VIC, Australia; ^5^Centre for Pharmacognosy and Phytotherapy, School of Pharmacy, University College London, London, United Kingdom; ^6^Central Ayurveda Research Institute Under Central Council for Research in Ayurvedic Sciences (Ministry of Ayush), Kolkata, India; ^7^Department of Endocrinology and Metabolism, All India Institute of Medical Sciences, Jodhpur, India; ^8^Institute of Applied Health Research, University of Birmingham, Birmingham, United Kingdom; ^9^West Bengal State Medicinal Plants Board, Kolkata, India; ^10^Sree Chitra Tirunal Institute for Medical Sciences and Technology, Trivandrum, India; ^11^Department of Endocrinology, Metabolism and Diabetes, All India Institute of Medical Sciences, New Delhi, India; ^12^Department of Non-communicable Disease Epidemiology, London School of Hygiene and Tropical Medicine, London, United Kingdom; ^13^Department of Kayachikitsa, J. B. Roy State Ayurvedic Medical College and Hospital, Kolkata, India; ^14^West Bengal Ayurvedic Practitioners Association (Paschimbanga Ayurved Chikitsak Samity), Kolkata, India

**Keywords:** development, clinical guideline, type 2 diabetes mellitus, Ayurveda, management

## Abstract

**Background:**

Type 2 diabetes mellitus (T2DM), a common chronic health condition, has major health and socioeconomic consequences. In the Indian subcontinent, it is a health condition for which individuals commonly consult Ayurvedic (traditional medical system) practitioners and use their medicines. However, to date, a good quality T2DM clinical guideline for Ayurvedic practitioners, grounded on the best available scientific evidence, is not available. Therefore, the study aimed to systematically develop a clinical guideline for Ayurvedic practitioners to manage T2DM in adults.

**Methods:**

The development work was guided by the UK’s National Institute for Health and Care Excellence (NICE) manual for developing guidelines, the Grading of Recommendations, Assessment, Development and Evaluation (GRADE) approach, and the Appraisal of Guidelines for Research and Evaluation (AGREE) II instrument. First, a comprehensive systematic review was conducted which evaluated Ayurvedic medicines’ effectiveness and safety in managing T2DM. In addition, the GRADE approach was used for assessing the certainty of the findings. Next, using the GRADE approach, the Evidence-to-Decision framework was developed, and we focused on glycemic control and adverse events. Subsequently, based on the Evidence-to-Decision framework, a Guideline Development Group of 17 international members made recommendations on Ayurvedic medicines’ effectiveness and safety in T2DM. These recommendations formed the basis of the clinical guideline, and additional generic content and recommendations were adapted from the T2DM Clinical Knowledge Summaries of the Clarity Informatics (UK). The feedback given by the Guideline Development Group on the draft version was used to amend and finalize the clinical guideline.

**Results:**

A clinical guideline for managing T2DM in adults by Ayurvedic practitioners was developed, which focuses on how practitioners can provide appropriate care, education, and support for people with T2DM (and their carers and family). The clinical guideline provides information on T2DM, such as its definition, risk factors, prevalence, prognosis, and complications; how it should be diagnosed and managed through lifestyle changes like diet and physical activity and Ayurvedic medicines; how the acute and chronic complications of T2DM should be detected and managed (including referral to specialists); and advice on topics like driving, work, and fasting including during religious/socio-cultural festivals.

**Conclusion:**

We systematically developed a clinical guideline for Ayurvedic practitioners to manage T2DM in adults.

## Background

The prevalence of diabetes mellitus, one of the most common chronic diseases, is increasing (1). Currently, one in 10 adults is living with the disease, and approximately 44% are undiagnosed (1). Around 90% of adults diagnosed with the disease have type 2 diabetes mellitus (T2DM), and a large population with T2DM is undiagnosed (1). It is a complex metabolic disorder with major health and socioeconomic consequences (1, 2). In T2DM, chronic hyperglycemia is related to macro- and micro-vascular complications and death (1, 2).

Ayurveda, a major traditional medical system, is in use for thousands of years in the Indian subcontinent, such as in Nepal and India (3, 4). In the public healthcare system, qualified and registered Ayurvedic practitioners are deployed, often as the lead clinical provider (5, 6). Ayurvedic practitioners also practice medicine in private clinics (5, 7). In Ayurveda, the corresponding term for diabetes mellitus is madhumeha, and the meaning of madhu is sweetness and meha is excessive urination (8, 9). In classical Ayurvedic texts, written in Sanskrit, this disease and its management have been described in detail (8, 9). Briefly, a multi-pronged and individualized approach is used to manage the disease, for example, through lifestyle modification (including diet) and Ayurvedic medicines (containing plant-, animal-, or mineral-origin ingredients–single or in combination). It is postulated that the mechanism of action includes pancreatic and extrapancreatic effects (8, 9). One of the leading diseases for which people visit Ayurvedic practitioners and use their medicines is T2DM, often uninterruptedly from the time of diagnosis (5, 6, 10–15). Ayurveda is commonly used by people with T2DM in the Indian subcontinent as it fits with their culture and health beliefs, and therefore, its acceptability, perceived relief, and satisfaction are generally high, particularly among older, poor, rural, and indigenous/minority people (13, 15, 16). Many individuals with T2DM do not prefer using western medicines because of the related side effects, administration mode (e.g., injections), and cost (12–15).

A lack of consistency can be seen in how Ayurvedic practitioners manage the disease, and many actions at the various stages of the care pathway (including screening for complications and referral to specialists) are mostly left to the individual Ayurvedic practitioner’s judgment, leading to unacceptable variations (11, 17, 18). They prescribe many non-evidence-based Ayurvedic medicines, which can have serious adverse effects on individuals, including heavy metal poisoning (19). They often follow claims made by peers or opt for an approach like “trial and error” (18, 20). One of the key hurdles highlighted by them is the lack of a good quality T2DM clinical guideline that can assist their clinical decision-making process and delivery of high-quality care to individuals (18).

High-quality T2DM clinical guidelines are effectively used in western medicine for improving the clinical care of individuals (21, 22). However, no such T2DM clinical guideline is available in Nepal for Ayurvedic practitioners, but such clinical guidelines are available in India (8, 9, 23–25). However, their quality is questionable due to several factors, including whether the best available scientific evidence was taken into consideration (26). Most of these clinical guidelines are short and limited in scope and have heterogeneous content with no clear recommendations for action at the different stages of the care pathway (26). Based on the Appraisal of Guidelines for Research and Evaluation II (AGREE II) instrument, which covers domains like scope and purpose of the guideline, stakeholder involvement, the rigor of development, clarity of presentation, applicability, and editorial independence (27), the overall quality of these clinical guidelines is poor, and these guidelines cannot be recommended for use in clinical practice (26). Poor quality clinical guidelines can lead to ineffective interventions’ usage and limited resources’ inefficient usage and can harm patients (28).

A good quality T2DM clinical guideline for Ayurvedic practitioners, grounded on the best available scientific evidence, may address the existing problems, discourage the use of Ayurvedic medicines of no, minimal, or questionable value, and encourage the use of effective and safe Ayurvedic medicines. Additionally, the clinical guideline may close the gap between what they do to manage T2DM and what the scientific evidence supports. Therefore, the study aimed to systematically develop a clinical guideline to manage T2DM in adults by qualified and registered Ayurvedic practitioners. The purpose is to provide them with a readily accessible summary of the current scientific evidence base and practical guidance on best practices for the management of T2DM.

## Methods

**FIGURE 1:**
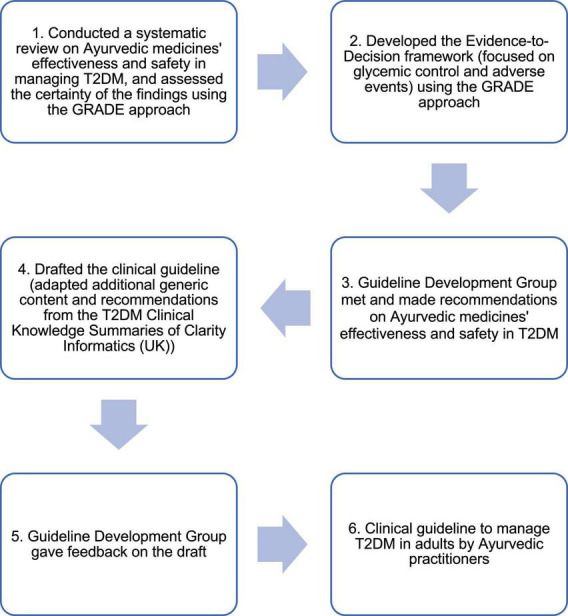
Systematic process to develop the clinical guideline.

We followed a systematic process for developing this clinical guideline as shown in Figure 1, and it was guided by the UK’s National Institute for Health and Care Excellence (NICE) manual for developing guidelines, the Grading of Recommendations, Assessment, Development and Evaluation (GRADE) approach, and the AGREE II instrument (27, 29, 30).

First, a comprehensive systematic review (and meta-analysis) was conducted to evaluate and synthesize the scientific evidence on Ayurvedic medicines’ effectiveness and safety in managing T2DM. The details are published elsewhere (31). Briefly, we followed the JBI systematic review methodology to ensure the findings from the review were scientifically robust (32). Randomized controlled trials (RCTs) assessing Ayurvedic medicines’ effectiveness and safety to manage newly diagnosed T2DM (treatment naïve) or existing cases of T2DM (on treatment) in adults (≥18 years) were included. RCTs were eligible if evaluated any classical or proprietary Ayurvedic medicine (e.g., containing plant- or mineral-origin ingredients–single or in combination) in any form (e.g., capsules, tablets, decoction, powder) with no intervention, placebo, non-pharmaceutical intervention (e.g., yoga), or pharmaceutical intervention (i.e., western oral antidiabetic drug or head-to-head comparison with another Ayurvedic medicine). The Ayurvedic medicines had to be administered for at least 8 weeks, and the timing of outcome measurements had to be at least 8 weeks from randomization. We conducted a comprehensive search of sources (including 18 electronic databases) till 16 January 2021 for finding published and unpublished RCTs. We developed the search strategies based on relevant previous systematic reviews and clinical guidelines and in consultation with an experienced information specialist. There were no language restrictions. We conducted data synthesis using random effects meta-analysis and reported pooled results as mean differences (MD) with 95% confidence intervals (CI). We used the GRADE approach for assessing the certainty of the findings–the findings were initially ranked as high (⊕⊕⊕⊕), and if there was serious evidence of the following five: risk of bias, inconsistency of results, indirectness of evidence, imprecision, and/or publication bias, the findings were downgraded to moderate (⊕⊕⊕○), low (⊕⊕○○), or very low (⊕○○○) (28). The review included 219 articles on 199 RCTs (21,191 participants), evaluating 98 Ayurvedic medicines. We conducted meta-analysis on 33 Ayurvedic medicines (including 32 single herbs), and the effects on glycemic control are shown in Table 1. Unfortunately, we could not include 65 Ayurvedic medicines in any meta-analysis, administered either as a single medicine or in combination with other Ayurvedic medicines, due to being evaluated in a single study. Few RCTs reported on adverse events, and if reported, adverse events were mostly none to mild, and mainly gastrointestinal tract related.

**TABLE 1:** Effects of Ayurvedic medicines on HbA1c and FBG.

Ayurvedic medicine compared to no medicine, no additional medicine, or placebo (unless mentioned otherwise)	HbA1c (%) MD; 95% CI (Number of RCTs)	FBG (mg/dl) MD; 95% CI (Number of RCTs)
*Aegle marmelos* (L.) Corrêa	**−1.6; −3 to −0.3** **(2 RCTs)**	**−56; −104 to −9** **(3 RCTs)**
*Allium sativum* L.	−0.4; −0.9 to 0.1 (3 RCTs)	−1; −14 to 11 (4 RCTs)
*Aloe vera* L.	−0.9; −2.1 to 0.3 (3 RCTs)	−11; −32 to 10 (4 RCTs)
*Anethum graveolens* L.	Meta-analysis not possible (<2 RCTs)	−12; −30 to 7 (2 RCTs)
*Azadirachta indica* A. Juss.	Meta-analysis not possible (<2 RCTs)	**−8; −13 to −4** **(2 RCTs)**
*Boswellia serrata* Roxb.	**−0.5; −0.7 to −0.4** **(2 RCTs)**	**−24; −28 to −21** **(2 RCTs)**
*Camellia sinensis* (L.) Kuntze	−0.1; −0,4 to 0.2 (6 RCTs)	−11; −26 to 5 (4 RCTs)
*Cinnamomum aromaticum* Nees	−0.2; −0.5 to 0.1 (10 RCTs)	1; −6 to 9 (9 RCTs)
*Cinnamomum verum* J. Presl	−0.1; −0.5 to 0.3 (6 RCTs)	**−11; −19 to −3** **(6 RCTs)**
*Citrullus colocynthis* (L.) Schrad.	−0.2; −0.7 to 0.4 (2 RCTs)	−3; −18 to 12 (2 RCTs)
*Coccinia grandis* (L.) Voigt	−0.5; −1.1 to 0 (2 RCTs)	**−22; −25 to −19** **(2 RCTs)**
*Crocus sativus* L.	0.2; −0.1 to 0.4 (4 RCTs)	−9; −26 to 8 (5 RCTs)
*Cuminum cyminum* L.	−1.5; −3.7 to 0.7 (2 RCTs)	−14; −35 to 6 (2 RCTs)
*Curcuma longa* L.	−0.2; −0.7 to 0.4 (6 RCTs)	**−10; −15 to −5** **(6 RCTs)**
*Cyamopsis tetragonoloba* (L.) Taub.	Meta-analysis not possible (<2 RCTs)	−7; −58 to 44 (2 RCTs)
*Elettaria cardamomum* (L.) Maton	0.2; −0.2 to 0.5 (2 RCTs)	1; −9 to 12 (2 RCTs)
*Enicostemma axillare* (Lam.) Raynal (versus oral antidiabetic drug)	Meta-analysis not possible (<2 RCTs)	23; −20 to 66 (2 RCTs)
*Gynostemma pentaphyllum* (Thunb.) Makino	**−1; −1.5 to −0.6** **(2 RCTs)**	**−29; −43 to −15** **(2 RCTs)**
*Ipomoea batatas* (L.) Lam.	−0.2; −0.5 to 0.1 (2 RCTs)	**−8; −13 to −3** **(2 RCTs)**
*Juglans regia* L.	−0.3; −0.6 to 0 (5 RCTs)	**−14; −24 to −4** **(5 RCTs)**
*Momordica charantia* L.	**−0.3; −0.4 to −0.1** **(7 RCTs)**	**−14; −23 to −4** **(7 RCTs)**
*Momordica charantia* L. (versus oral antidiabetic drug)	0.4; 0.2 to 0.7 (2 RCTs)	14; 9 to 19 (2 RCTs)
*Nigella sativa* L.	**−0.4; −0.6 to −0.1** **(4 RCTs)**	−15; −30 to 0 (7 RCTs)
*Plantago ovata* Forssk.	**−0.9; −1.4 to −0.3** **(3 RCTs)**	**−32; −40 to −23** **(3 RCTs)**
*Portulaca oleracea* L.	Meta-analysis not possible (<2 RCTs)	−10; −34 to 14 (3 RCTs)
*Pterocarpus marsupium* Roxb. (versus oral antidiabetic drug)	Meta-analysis not possible (<2 RCTs)	16; −7 to 39 (2 RCTs)
*Punica granatum* L.	−0.1; −0.5 to 0.4 (6 RCTs)	−8; −16 to 1 (6 RCTs)
*Sesamum indicum* L.	−0.7; −1.4 to 0 (2 RCTs)	−46; −116 to 25 (2 RCTs)
Shilajit	−0.3; −0.7 to 0.2 (2 RCTs)	Meta-analysis not possible (<2 RCTs)
*Syzygium cumini* (L.) Skeels	−0.1; −1.5 to 1.3 (2 RCTs)	−5; −40 to 29 (2 RCTs)
*Tinospora cordifolia* (Willd.) Hook. f. and Thomson	**−0.5; −0.6 to −0.5** **(2 RCTs)**	**−4; −6 to −3** **(2 RCTs)**
*Trigonella foenum-graecum* L.	**−0.6; −0.9 to −0.4** **(12 RCTs)**	**−14; −22 to −5** **(13 RCTs)**
*Trigonella foenum-graecum* L. (versus oral antidiabetic drug)	0.3; −1 to 1.6 (2 RCTs)	27; −24 to 79 (2 RCTs)
*Urtica dioica* L.	**−1.3; −2.4 to −0.2** **(3 RCTs)**	−20; −41 to 1 (8 RCTs)
*Zingiber officinale* Roscoe	−0.3; −0.6 to 0.1 (9 RCTs)	−8; −17 to 1 (8 RCTs)

Results reported in bold are statistically significant at the 5% level.

Next, the GRADE approach was used to develop the Evidence-to-Decision framework for each of the 33 Ayurvedic medicines, and we focused on glycemic control and adverse events (28). A societal perspective (including all costs, regardless of who pays) was taken, and the following domains were considered in the Evidence-to-Decision framework: (i) Is the problem a priority? (ii) How substantial are the desirable anticipated effects? (iii) How substantial are the undesirable anticipated effects? (iv) What is the overall certainty of the evidence of effects? (If the quality of the scientific evidence was the same for glycated hemoglobin (HbA1c) and fasting blood glucose (FBG), then this became the overall quality of evidence, but if it differed across HbA1c and FBG, then the lowest quality of evidence for these became the overall quality of evidence.) (v) Is there important uncertainty about or variability in how much people value the main outcomes? (vi) Does the balance between desirable and undesirable effects favor the intervention or the comparison? (vii) Is the intervention acceptable to key stakeholders? (viii) Is the intervention feasible to implement?

A Guideline Development Group was formed, which comprised 17 internationally based members consisting of Ayurvedic practitioners, western medicine practitioners (including diabetologists), pharmacognosists and experts in medicinal plants and phytochemistry, systematic review methodologists, medical statisticians, epidemiologists, sociologists, and people with T2DM. The direct and indirect interests of the members were considered, but no potential conflicts of interest were identified. A Delphi consensus-based technique was used to develop the recommendations (along with justification), using the scientific evidence presented in the Evidence-to-Decision framework (and their expertise and/or experience) (28). The following recommendations were available: a strong recommendation for or against the intervention (i.e., Ayurvedic medicine), a conditional recommendation for or against the intervention (i.e., weak recommendation), or no recommendation/recommendation to use the intervention only in research. A strong recommendation was given where a high or moderate overall quality of evidence was reported, and the wording of the recommendation included either “offer” (↑↑) or “do not offer” (↓↓), depending on the direction (for or against, respectively). A weak recommendation was given where a low or very low overall quality of evidence was reported, and the wording of the recommendation included either “consider offering” (↑?) or “do not consider offering” (↓?), depending on the direction (for or against, respectively). In the systematic review, we found that the daily doses of Ayurvedic medicines varied in the included RCTs, depending on their type and form and timing of administration (31). Therefore, the Guideline Development Group considered the information available in the systematic review before providing recommended doses for the different Ayurvedic medicines.

The recommendations from the Guideline Development Group on Ayurvedic medicines’ effectiveness and safety in T2DM were used to form the basis of the clinical guideline, and additional generic content and recommendations were adapted from the T2DM Clinical Knowledge Summaries (June 2021 version; accredited by NICE) of the Clarity Informatics (UK) with permission (33). This approach was taken because the basic principle of T2DM management is the same in western and Ayurvedic medical systems i.e., a combination of a healthy lifestyle and medicinal products (8, 9, 33); however, the scientific evidence base is extremely limited for components like the Ayurvedic lifestyle.

Finally, the draft version of the clinical guideline was shared with the Guideline Development Group. Experts mainly gave feedback on the applicability and feasibility of the content and readability and understandability of the clinical guideline, and people with T2DM primarily provided their views and preferences. The feedback was used to amend and finalize the clinical guideline.

## Results

A clinical guideline for managing T2DM in adults by Ayurvedic practitioners was developed, which focuses on how practitioners can provide appropriate care, education, and support for people with T2DM (and their carers and family). Table 2 summarizes the broad topics covered in the clinical guideline. Briefly, it provides information on T2DM, such as its definition, risk factors, prevalence, prognosis, and complications; how it should be diagnosed and managed through lifestyle changes like diet and physical activity and Ayurvedic medicines; how the acute and chronic complications of T2DM should be detected and managed (including referral to specialists); and advice on topics like driving, work, and fasting including during religious/socio-cultural festivals. The following issues are outside the scope of the clinical guideline: people with other types of diabetes or at high risk of developing T2DM; women with T2DM who are pregnant, planning a pregnancy, or breastfeeding; management of T2DM using western medicines or insulin; and detailed recommendations on the management of comorbidities, emergencies, and complications related to T2DM.

**TABLE 2:** Broad topics covered in the clinical guideline.

Section	Topics included
Background information	What is T2DM?
	What causes T2DM?
	What are the risk factors for T2DM?
	How common is T2DM?
	What is the prognosis of T2DM?
	What are the complications of T2DM?
Diagnosis	When should I suspect T2DM in an adult? *Interpreting HbA1c results*
	When should I suspect a hyperglycemic emergency? *Precipitating factors*
	When should I suspect hypoglycemia in a person with T2DM?
Management	What initial information and advice should I offer a person with T2DM?
	What are the treatment targets for people with T2DM?
	Which Ayurvedic antidiabetic medicines are available for people with T2DM?
	What lifestyle advice should I give to people with T2DM? *Diet* *Exercise and physical activity* *Alcohol intake* *Smoking and drug misuse*
	How should I screen for and manage complications in people with T2DM? *Retinopathy* *Foot problems* *Diabetic kidney disease* *Cardiovascular risk factors* *Peripheral and autonomic neuropathy*
	How should I manage a person with a suspected hyperglycemic emergency?
	How should I manage intercurrent illness in a person with T2DM? *“Sick-day rules”*
	How should I manage hypoglycemia in a person with T2DM? *Recognizing hypoglycemia* *Managing a person with impaired awareness of hypoglycemia* *Managing an acute episode of hypoglycemia* *Managing nocturnal hypoglycemia* *Preventing hypoglycemic episodes*
	What additional information and advice should I give people with T2DM? *Advice on driving* *Advice on insurance* *Advice on fasting, including religious/socio-cultural festivals* *Advice on work* *Advice on holidays and travel*

## Discussion

We report the systematic development process of a clinical guideline to manage T2DM in adults by Ayurvedic practitioners. Ayurvedic practitioners based in the Indian subcontinent, such as Nepal and India, may find this clinical guideline useful due to many similarities, such as in the population, context, and setting. The clinical guideline may also be highly relevant in countries with South Asian ethnic minorities who often rely heavily on such treatments (34, 35), as the guideline is based on a systematic review of the worldwide literature.

There is a need to evaluate clinical guidelines and scale these up if found to be effective in improving outcomes (27, 36, 37). In this case, the evaluation is specifically important, as we used an innovative approach to integrate Ayurvedic and western medical systems. Here, integration does not mean the prescription of western medicines by Ayurvedic practitioners (and vice-versa) but focusing on management issues like generic advice given to patients and referral to specialists for complications. Therefore, a feasibility cluster RCT is currently in progress in Nepal to determine the feasibility of undertaking the definitive cluster RCT (38). As part of the feasibility study, we will also be conducting semi-structured interviews with Ayurvedic practitioners to explore their acceptability of the clinical guideline and factors that can facilitate or impede its uptake and adherence. If the feasibility of undertaking the definitive cluster RCT is promising, the definitive trial will be conducted for determining the effectiveness of the intervention. If it is found to be effective, individuals with T2DM will have improved health outcomes, such as better blood glucose control and lower T2DM complications. The related future clinical, personal, and economic burden on individuals with T2DM, their carers and family, and the health system and the economy will be reduced. Individuals with T2DM will be cared for in line with the best available scientific evidence and in the same way irrespective of where or by which Ayurvedic practitioner they are treated.

To the best of our knowledge, this was the first time a systematic process was followed to develop a clinical guideline for Ayurvedic practitioners. The process was guided by some of the best guideline development methods and manuals. A significant challenge was to systematically integrate Ayurvedic and western medical systems for managing T2DM, and the best available scientific evidence on effective and safe Ayurvedic medicines in T2DM was used. We also involved a range of experts and people with T2DM, and the process helped us to reach a consensus on such a complex health intervention. If both Ayurvedic and western medical systems work together for the better management of T2DM, it will reduce the large gap in the doctor-patient ratio in South Asian countries (39). As we are evaluating the intervention (i.e., the clinical guideline) and the evaluation will take a number of years to complete, we are unable to publish the complete clinical guideline at this stage to avoid contamination in the control group. However, we have published its development process so that similar approaches could be used to develop clinical guidelines for managing other health conditions by Ayurvedic practitioners or in other traditional medical systems throughout the world. Clinical guidelines should be developed to reflect current research. At the time of the development of the clinical guideline, the comprehensive systematic review from which the evidence was considered was up to date (last searched to 16 January 2021). However, it would be prudent to consider updating the searches of the systematic review to capture more recent evidence before the clinical guideline is implemented more widely (and should include other essential aspects, such as monitoring and auditing criteria to measure the application of guideline recommendations). There is uncertainty around issues like the interaction of Ayurvedic and western medicines, which needs more research before making any recommendation in the clinical guideline. We only considered Ayurvedic medicines for which there was evidence of effectiveness from a comprehensive systematic review and meta-analysis of RCTs (31), which will mean that some potentially effective interventions may have been missed from either not having been tested in an RCT or those which could not be included in any meta-analysis due to being evaluated in a single RCT.

In conclusion, we systematically developed a clinical guideline for Ayurvedic practitioners to manage T2DM in adults. A feasibility cluster RCT is in progress.

## Data availability statement

The original contributions presented in this study are included in this article/supplementary material, further inquiries can be directed to the corresponding author.

## Ethics statement

Ethics approval was obtained from the Research Ethics Committee, Faculty of Medicine and Health Sciences, University of Nottingham, UK (511-2003). Written informed consent for participation was not required for this study in accordance with the national legislation and the institutional requirements.

## Author contributions

KC conceptualized and designed the clinical guideline development process with the help of MH, SL, SG, PJ, NT, SK, TB, and JL-B. KC developed the clinical guideline with the help of other authors (i.e., the Guideline Development Group comprising NK, MH, MM, AM, SL, SG, SM, IP, PJ, NT, SK, TB, JL-B, and people with T2DM). KC wrote the first draft of the manuscript. All authors contributed significantly to the revision of the manuscript and read and approved the final manuscript.
